# Cryptic female Strawberry poison frogs experience elevated predation risk when associating with an aposematic partner

**DOI:** 10.1002/ece3.2662

**Published:** 2016-12-24

**Authors:** Julia Carolina Segami Marzal, Andreas Rudh, Björn Rogell, Anders Ödeen, Hanne Løvlie, Charlotte Rosher, Anna Qvarnström

**Affiliations:** ^1^ Animal EcologyDepartment of Ecology and GeneticsUppsala UniversityUppsalaSweden; ^2^ Department of Zoology: EcologyStockholm UniversityStockholmSweden; ^3^ IFM BiologyLinköping UniversityLinköpingSweden; ^4^ Faculty of Life SciencesUniversity of ManchesterManchesterUK

**Keywords:** aposematism, assortative mating, crypsis, *Oophaga pumilio*, predation, sexual selection, speciation

## Abstract

Population divergence in sexual signals may lead to speciation through prezygotic isolation. Sexual signals can change solely due to variation in the level of natural selection acting against conspicuousness. However, directional mate choice (i.e., favoring conspicuousness) across different environments may lead to gene flow between populations, thereby delaying or even preventing the evolution of reproductive barriers and speciation. In this study, we test whether natural selection through predation upon mate‐choosing females can favor corresponding changes in mate preferences. Our study system, *Oophaga pumilio*, is an extremely color polymorphic neotropical frog with two distinctive antipredator strategies: aposematism and crypsis. The conspicuous coloration and calling behavior of aposematic males may attract both cryptic and aposematic females, but predation may select against cryptic females choosing aposematic males. We used an experimental approach where domestic fowl were encouraged to find digitized images of cryptic frogs at different distances from aposematic partners. We found that the estimated survival time of a cryptic frog was reduced when associating with an aposematic partner. Hence, predation may act as a direct selective force on female choice, favoring evolution of color assortative mating that, in turn, may strengthen the divergence in coloration that natural selection has generated.

## 1 Introduction

Over recent decades, it has become well established that natural selection and sexual selection, rather than neutral processes, are the main drivers of population divergence and speciation (Butlin, Bridle, & Schluter, 2009; Coyne & Orr, 2004; Nosil, 2012; Price, 2008; Schluter, 2001; Schluter & Conte, 2009). Nevertheless, many of the mechanisms by which natural and sexual selection can interact during the speciation process remain largely unexplored. One major argument why sexual selection through female mate choice may often counteract population divergence is based on the assumption that mate preferences often are directional and open‐ended toward males with larger or brighter ornaments (Ritchie, 2007). In this study, we test a simple but previously overlooked mechanism (i.e., direct selection arising through predation that disfavors females from the more cryptic populations that mate dis‐assortatively), which could solve this “problem”.

Sexually selected signals are often not only conspicuous to potential mates, but also to predators (Burk, 1982; Darwin, 1871; Endler, 1992). The evolution of many sexually selected traits is therefore often driven toward optimal compromises between attractiveness to potential sexual partners and avoidance of being conspicuous to predators (Andersson, 1994; Tuttle & Ryan, 1981; Zuk & Kolluru, 1998). It is well known that variation in predation pressures across environmental conditions experienced by different populations of the same species therefore can lead to rapid adaptive population divergence in coloration, as, for example, demonstrated in Trinidadian guppies, *Poecilia reticulata* (Endler & Houde, 1995; Magurran, Seghers, Shaw, & Carvalho, 1995; Labonne & Hendry, 2010). However, directional and open‐ended mate preferences (i.e., favoring the most conspicuous males) may lead to considerable gene flow between populations and thereby delay or even prevent evolution of reproductive barriers. As mate‐choosing females may also face increased predation risk, females are often expected to adjust their degree of choosiness according to the degrees of predation pressures (Maan & Seehausen, 2011). Variation in female choosiness may thus lead to differences in the strength of sexual selection acting on male display across populations, but differences in female choosiness will not ensure population assortative mating at secondary contact (because the direction of choice toward the most conspicuous males remains). A crucial question then becomes whether natural selection instead of favoring relaxed choosiness may act specifically against individuals that choose to mate with conspicuous partners, thereby increasing the proportion of individuals that prefer less conspicuous mates in the population. This situation would allow divergence in preferences between populations and would also ensure assortative mating at secondary contact. However, the fact that natural selection, in the form of predation, could also directly affect the evolution of specific mate preferences remains underexplored.

The neotropical poison dart frog *Oophaga pumilio* (family Dendrobatidae) has become an established model system for research on speciation (Maan & Seehausen, 2011). This small diurnal frog has a distribution range from Nicaragua to central Panama and is one of the most color polymorphic Dendrobatids with more than 18 different described color morphs (Qvarnström et al., 2014; Rudh, Rogell, & Höglund, 2007; Summers, Cronin, & Kennedy, 2004). Most of this diversity is found in a small range of its distribution in the islands of the Bocas del Toro Archipelago, Panamá. Like all poison dart frogs, these different populations of *O. pumilio* are highly toxic, but are unique in that they generally use two ultimately different strategies against predators: aposematism and crypsis. Aposematism is an antipredator strategy that relies on conspicuous signals to warn predators about the unpalatability of the prey. The evolution of exaggerated traits improving mating success should thereby be relieved from many of the usually imposed constraints associated with increased risks of predation (Rudh, Rogell, Håstad, & Qvarnström, 2011). By contrast, the cryptic strategy relies on the ability of the individuals to remain unnoticed to predators, and frog populations with less aposematic coloration are also observed to be slightly less toxic (Maan & Cummings, 2012).

Some of the populations of Bocas del Toro are not completely geographically isolated from each other (Maan & Cummings, 2008). Color is an important trait for mate choice in this species (Maan & Cummings, 2008, 2009; Richards‐Zawacki & Cummings, 2011; Richards‐Zawacki, Wang, & Summers, 2012) and females tend to prefer their own color morph, at least in captivity (Reynolds & Fitzpatrick, 2007; Summers, Symula, Clough, & Cronin, 1999), while this is not always the case in nature (Meuche, Brusa, Linsenmair, Keller, & Prohl, 2013). In some populations, females have been found to prefer more brightly colored males per se (Maan & Cummings, 2009). Male *O. pumilio* frogs hold territories where they call to attract females and mating success correlates with calling activity and average perch height (Pröhl & Hödl, 1999). Males are highly intrasexually competitive (Pröhl, 2005), and females, at least partly, choose their mates based on tadpole‐rearing sites within the defended territory (Pröhl & Berke, 2001). Aposematic frogs are more aggressive and explorative (Rudh, Breed, & Qvarnström, 2013) and use more exposed calling sites (Rudh et al., 2011). Thus, there are several reasons why aposematic males may have a mating advantage as opposed to cryptic males in populations where both strategies co‐occur.

In this study, we investigated whether predation could act directly on females as a selective force disfavoring cryptic females that mate disassortatively with regard to color morph (i.e., females that are attracted to conspicuously colored aposematic males). In short, we base our experimental setup on the two following general assumptions: (1) Predators prefer to attack and eat cryptic prey over prey that signals unprofitability (here aposematism) (Darst & Cummings, 2006; Ruxton, Sherratt, & Speed, 2004) and (2) highly ornamented or brightly colored males (including aposematic males) have a mating advantage simply because they are more easy to find by females or they may defend better territories or provide other benefits and/or they deter rivals (Andersson, 1994). Taking these two well‐established assumptions together, the evolution of a preference for aposematic males among aposematic females is expected to happen and is not difficult to explain (either in allopatry or sympatry). However, the evolution of a preference for cryptic males among cryptic females (and not only a more relaxed degree of choosiness in response to a higher predation pressure) is more difficult to explain. Why should cryptic females prefer the less colorful cryptic males in color polymorphic populations? We hypothesize that selection resulting from predation acting directly on females as they select their mate and associate with males can explicitly disfavor cryptic females that mate color disassortatively. More specifically, we test whether the presence and proximity of an aposematic frog increases the speed by which the perceived edible cryptic female is caught. In order to simulate a scenario of an avian predator attack, we first trained female domestic fowl (*Gallus gallus domesticus*) to find digitalized images of cryptic frogs against a natural forest background on a computer screen. The domestic fowl was chosen as a model because they represent a typical avian predator (Darst, Cummings, & Cannatella, 2006; Maan & Cummings, 2012; Qvarnström et al., 2014). Second, we simulated predation trials where fowls were exposed to images of either a single cryptic frog, or a cryptic frog together with an aposematic frog at three different distances from the focal frog to mimic the situation when a cryptic frog approaches an aposematic partner. Our main aim was to use this experimental approach to test whether the survival probability (measured as the time it took the predator to localize the cryptic frog) is reduced when the cryptic frog approaches an aposematic mate.

## 2 Methods

We performed an experiment in which female fowl were trained to find digitized images of cryptic frogs on a computer screen. To test different treatments, we created a computer simulation based on spectrum measurements, pictures of *Oophaga pumilio* individuals, and pictures of the natural substrate background. The experiment was carried out between June and July of 2015 at Tovetorp research station (Södermanland, Sweden, Stockholm University). Photographs, irradiance, and reflectance spectra data were the same as those used by Qvarnström et al. (Qvarnström et al., 2014). Likewise, we calibrated a 22‐inch Samsung S22C200 computer screen to match the relative quantum catch by chicken cone photoreceptors with a pairwise difference of <6% (see supplementary material, Håstad & Ödeen, 2008; Qvarnström et al., 2014).

### 2.1 Computer simulation

**Figure 1:**
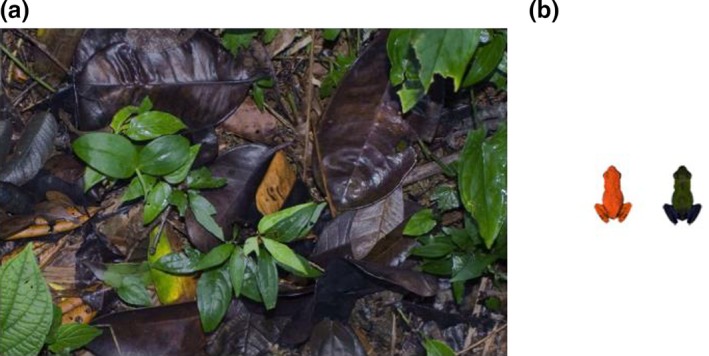
(a) Image of the natural background of rainforest soil used in the experiment, and (b) images of an aposematic frog (left) and a cryptic frog (right) presented to the hens during experimental trials

The treatments were based on images showing a single green cryptic frog (here called “control”) or a cryptic frog together with a red aposematic frog (“mixed‐pair couple”), on a natural background of soil with a mix of brown and green leaves (Figure 1a). The two frog types (“cryptic” and “aposematic”) were photographs of two naturally occurring morphs of *O. pumilio* (Figure 1b), and they were showed on the screen in an average natural size of 17 mm (Pröhl, Hagemann, Karsch, & Höbel, 2007). The program generated unique images by presenting the background randomly rotated and with the two frog images each being rotated a random number of degrees and each being placed at a random position on the image. The mixed couple of frogs was separated by three different fixed distances from each other (1–2 cm “short”, 3–4 cm “intermediate”, and 7–10 cm “long”). Thus, there were four different treatments in total (Table 1). The latency until the cryptic frog was found by the predator was recorded (“catch time”), and the image was changed between each time the prey was found by the predator. The images were shown on the screen using a script written in Java language and run in the Eclipse Java Environment.

**Table 1:** Experimental treatments, that is, digital images of cryptic frogs presented at different distances from an aposematic partner to fowls trained at finding cryptic frogs and number of data points obtained per treatment. The control treatment consisted of a single cryptic frog shown on the screen

Treatment	Distance (cm)	*N*
Short	1–2	205
Intermediate	3–4	213
Long	7–10	211
Control	NA	631

### 2.2 Domestic fowl population and training

We used female domestic fowl from a population of an old Swedish game breed of fowl (“Gammal svensk dvärghöna”). These birds are behaviorally and morphologically very similar to the wild ancestor of all domesticated chickens, the red jungle fowl (*Gallus gallus*) (see references in, e.g., Favati, Leimar, & Løvlie, 2014). The birds are kept under semi‐natural conditions in outdoor aviaries and are frequently used in similar behavioral experiments (e.g., Lisney et al., 2011; Qvarnström et al., 2014). Only birds younger than 4 years old were used, to minimize any possible age‐related effects on the vision of the birds.

Hens (*n* = 20) were trained individually to recognize and peck on the image of a cryptic frog by using boiled spaghetti as a reward. The experiment was carried out indoors using a skinner box (50 × 60 × 60 cm) (for details, see supplementary material). A peck was defined as successful when the hen pecked on the screen inside the area of the contour of the frog making a clear sound at the contact with the screen. Latency until the hen pecked on the green frog (“catch time”) was recorded with a manual chronometer. Each of these was considered a trial, which had a maximum duration of 120 s after which the experiment was set to proceed with a consecutive trial. If the hen started to become unfocused on the task or tried to jump out of the skinner box, the session (which consisted of consecutive trials) was interrupted; this usually happened first after about an hour of continuous experimentation.

The first step of the training consisted of teaching each hen individually to recognize and peck at cryptic, green frog images on a white background. Once this was achieved, the aposematic frog was showed at the same time as the cryptic one, but the hen was only rewarded when the peck was on the cryptic frog. When the hen had learned the association of the reward with the green frog, determined by pecking on the correct frog over five consecutive trials, the background saturation was progressively increased from 0% to a 100% in four stages (Figure S2). The training was considered complete when a hen pecked on the correct frog with a 100% saturated background, over five consecutive trials. In general, birds needed between two and three sessions, each consisting of 50–60 trials lasting around an hour, to finish the training process.

We had four treatments in total, that is, a sole cryptic frog on the screen (“control”) or a “mixed‐pair couple” with three different distances (“short,” “intermediate,” and “long,” see computer simulation above) between the two individuals in the couple (Table 1). The treatments were presented to the hens in a random order. For each treatment, we tested 10 ± 1 data points (i.e., 10 trials for each treatment for each bird) resulting in a total of 1,260 data points (Table 1).

### 2.3 Statistical analyses

Statistical analyses were carried out in R version 3.1.0. (R Core Team, 2015). Because of the nature of the data and our research question, the most appropriate approach was to do a survival analysis. To estimate the survival probability of the green frog under the different treatments, we used a proportional hazards model fitted by maximum likelihood (R package “coxme”). The time it took the hens to find and peck on the frog (“catch time”) was used as the response variable, the four treatment groups as a fixed effect, and the identity of each hen as a random effect. The assumption of the proportional hazard was tested using the cox.zph function and was also visually verified by a log–log plot. To illustrate the results of the model, a treatment‐specific Kaplan–Meier curve was produced (R package survival). Finally, to verify the significance of the fixed effect, we compared models with and without the treatment effect, using likelihood ratio test.

## 3 Results

All hens completed the task in a satisfactory way and completed all required trials resulting in a total of 1,260 data points (Table 1). Even though the hens were trained to peck on the cryptic frog, they occasionally pecked on the aposematic frog during the trials. This event was extremely rare (three of 1,260), so its effect was regarded negligible.

**Figure 2:**
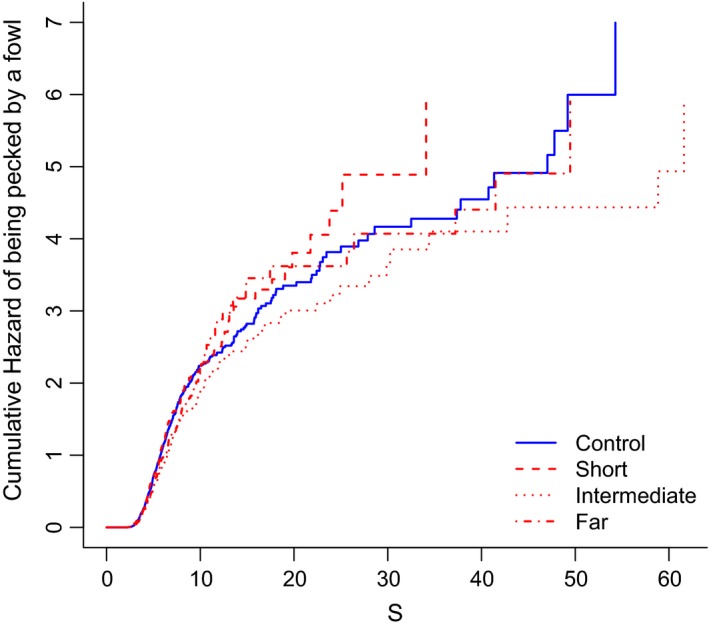
Kaplan–Meier curve of the Cox model from the proportional hazards model analysis. The graph illustrates the results of the proportional hazards model performed to analyze the survival probability of the frogs under different treatments (see Table 1). The variable under analysis was “catch time”, which represents the time passed until the predation event. More specifically, the figure shows on the *y*‐axis the probability of dying events in time (in seconds, on the *x*‐axis) depending on the treatments. The safest treatment is the “intermediate”, whereas the “short” treatment is the most dangerous

The latency of a hen to peck on the focal cryptic frog (i.e., the frog's “survival time”) was dependent on the treatment (χ^2^ = 8.28, *df* = 3, *p* = .04). The cryptic frog was found significantly faster by the hens when this frog was shown at a short distance from an aposematic frog (“short treatment”) as compared to shown alone on the screen (“control treatment”, Table 2). The cryptic frog was also found significantly faster by the hens when this frog was shown alone on the screed (“control treatment”) than when it was shown at an intermediate distance from an aposematic frog (“intermediate treatment”, Table 2). The coefficients of our proportional hazards model (Table 2) are estimates of the level of Hazard where a positive coefficient indicates an increase in the hazard (i.e., a reduction of the survival probability). We can therefore conclude that the most hazardous scenario for the cryptic frog was when this frog was shown at a short distance (“short treatment”) from an aposematic frog. The most secure scenario for the cryptic frog was appearing at an intermediate distance (“intermediate treatment”) from an aposematic frog (Figure 2). There was no apparent difference in the speed by which the chickens predated on the cryptic frog when this frog was shown alone on the screen (“control treatment”) or at a long distance from an aposematic frog (“long distance treatment”, Figure 2).

**Table 2:** Fixed effects coefficients of the proportional hazard model on the variable catch time (the latency of time an individual takes to peck on the focal cryptic frog). The coefficients are contrasted with the intermediate treatment (see Table 1); significant values are highlighted in bold

Fixed effects	Coefficient	exp (coef)	*SE* (coef)	*z* Value	*p* Value
Control	0.19	1.21	0.08	2.36	**.02**
Short	0.23	1.26	0.10	2.3	**.02**
Long	0.07	1.07	0.10	0.72	.47

Random effect	Variable	*SD*	Variance
ID	Intercept	0.45	0.20

## 4 Discussion

We used an experimental approach where domestic fowl predated on digitized images of cryptic frogs against a natural habitat background, to investigate the role of natural selection in shaping female mate choice preferences. The survival probability of cryptic frogs under this setup was reduced when the focal frog was close to an aposematic partner, compared to when the focal frog was alone. Our results therefore demonstrate that when a cryptic frog is close to an aposematic frog, as is the case when courting and mating, predators more easily find the former. As expected, the presence of an aposematic frog on the screen did not increase the risk of predation of the cryptic frog when the two frogs where shown at a further distance from each other. The time required for the predator to catch the cryptic frog when it was shown at a long distance from the aposematic frog was very similar to the when there was only one cryptic frog shown on the screen (our control treatment).

Somewhat counter‐intuitively, the intermediate distance between the cryptic and the aposematic frogs appeared to be the safest treatment for the cryptic frog (i.e., safer than being presented alone on the screen). It is possible that the aposematic frog attracted the attention of the predator, but that the cryptic frog was immediately outside a spatially acute foveal area of the predator's visual field. When the predator in the next step looked elsewhere in search for the cryptic frog, it is possible that a “safe” buffer zone was generated immediately onside the previous acute area of vision. Hence, the predator could have perceived this previous visually acute area (and accidentally an area immediately outside of it) as being empty of palatable frogs. Regardless of the underlying explanation to why the intermediate distance was the safest one, the fact that the short distance was the most dangerous one means that cryptic individuals that are being courted by or mating with aposematic individuals are selected against. Under natural conditions, courting and mating frogs stay very close to each other under a prolonged period and are often vocalizing at the same time (Limerick, 1980). Moreover, aposematic and cryptic frogs differ in their display behavior with aposematic individuals being more aggressive (Rudh et al., 2013) and using more exposed calling sites (Rudh et al., 2011). The underlying reason for a positive covariance between bright aposematic coloration, more aggressive and explorative behavior, and the use of more exposed calling sites is likely to be that aposematic males are released from the bound of predation as predators perceive them as unprofitable prey (Rudh et al., 2011). Our experimental results may therefore in fact be underestimating the increased risk of predation that cryptic frogs experience by being close to an aposematic individual. In addition, the well‐known “dilution effect” (Bertram, 1978; Treisman, 1975; Turner & Pitcher, 1986) means that cryptic females that associate with cryptic males should experience reduced risks of predation for yet another reason. Once the pair is detected by the predator, the two cryptic individuals constitute two possible targets for the predator, which reduces the chance that the female will be the prime target.

Females are in general assumed to become vulnerable to predation when they expose themselves during mate searching and assessment or due to mating itself (Lima & Dill, 1990). Previous experimental studies have shown that females often respond to an elevated predation risk by decreasing choosiness, resulting in a higher degree of random mating (Atwell & Wagner, 2015; Bonachea & Ryan, 2011; Godin & Briggs, 1996). In populations that are consistently exposed to higher levels of predation, lower levels of choosiness are expected to evolve as the choosiest individuals are selected against. As female choice is an important source of sexual selection (Andersson, 1994), local variation in predation pressures can hence limit the level of exaggeration of sexual signals both through effects on males (i.e., increased strength of natural selection against exaggeration) and females, (i.e., decreased choosiness that leads to weaker sexual selection favoring exaggeration in males). In this study, we instead tested whether there was an elevated risk of predation resulting directly from association with a conspicuous male per se, which has the potential to favor an evolutionary change in the direction of choice (i.e., cryptic females are selected to actively avoid conspecific males). A parallel divergence in male sexually selected traits and female choice (instead of just weaker choice in favor of aposematic males among cryptic females) has the potential to ensure population assortative mating at secondary contact.

Our experimental setup was based on field data, spectrum measurements, and pictures of *O. pumilio* individuals collected from populations in Bocas del Toro. Reproductive isolation is incomplete in this system, meaning that current interactions between natural and sexual selection may play a central role for the future prospects of this initiated speciation process. There are currently highly color polymorphic populations in, for example, the Bastimentos Island (Richards‐Zawacki & Cummings, 2011) and C. Brujo (pers. obs). The increased predation risk experienced by cryptic females that associate with aposematic males that we identify in this experimental study may lead to divergence in mate choice when cryptic and aposematic populations of this species experience secondary contact. The identified selection pressure may also have been important during the preceding evolutionary shifts from aposematism to crypsis that have occurred in these populations. We moreover argue that a predation cost imposed on females that prefer conspicuous partners may, in general, be a simple but overlooked mechanism that may lead to assortative mating between populations that have experienced different degrees of predation during periods of allopatric divergence. We predict that that this selective mechanism is particularly likely to be important when the predator prefers the female as a prey over the male. In the case of *O. pumilio* frogs, conspicuous aposematic individuals “catch the attention” of predators, but are not perceived as a palatable prey, which impose a particular high risk on cryptic individuals approaching aposematic partners. However, the same situation may apply to many other species where conspicuous males “catch the attention” of the predator that may nevertheless prefer females as prey.

There may be conspicuous sexual signals that are used by females to predict mate quality in many species (Andersson, 1994), making it possible that females are faced with a trade‐off between ensuring mate quality and safe mating. A potential evolutionary solution to this dilemma is that females direct their attention to alternative display traits that are not conspicuous to the most common predators in the environment rather than developing a preference for less ornamented and presumably low‐quality males. Evolution of female choice favoring alternative male display traits in populations experiencing differences in predation pressure may also result in population assortative mating driven by direct selection through predation on mate‐choosing females. Most studies on the evolution of multiple or alternative mate choice cues focus on costs and benefits that females experience *after* they have made their choice (Candolin, 2003). Increased predation pressure associated with male conspicuousness is in fact often considered to ensure honest signaling by the males and therefore translate into material or genetic benefits to females following the choice of conspicuous males (Andersson, 1994). If an increased predation risk associated with conspicuousness directly “spillover” to the female as demonstrated in our experimental study, the situation may become different. Under such a scenario, male sexual signals and female choice may diverge in parallel as a direct consequence of variation in the risk of predation across environments. We predict that natural selection through predation could favor females that have sensory characteristics that make them prefer to approach and mate with males that are less conspicuous to locally common predators. As the “spillover” risk of predation on the female increases with the time spent together with the male, this effect may have contributed to the general pattern with lower levels of sexual signaling among males in species with longer pair bounds.

In conclusion, our results imply that it is disadvantageous for a cryptic individual to associate with a conspicuous individual, which is the case during courting and mating, because of an increased probability of being found by a predator. Direct selection on female choice through predation therefore has the potential to drive evolution of assortative mating between populations using different antipredator strategies of the species *O. pumilio* in the archipelago of Bocas del Toro. We suggest that variation in predation across local environments inhabited by different populations of a species may in general not only affect the evolution of male sexual signals, but also directly act as a selective force on female mate preferences such that females with preferences for males with less conspicuous signals are favored by selection in predator‐dense environments.

## Conflict of Interest

None declared.
