# Supplementary material for: Cryptic female Strawberry poison frogs experience elevated predation risk when associating with an aposematic partner
Source: Ecol Evol. 2016 Dec 24;7(2):744–50. doi: 10.1002/ece3.2662 (PMC5243780; doi:10.1002/ece3.2662)
Supplement: Supplementary file 1 [file ECE3-7-744-s001.docx]

**Supplementary Material to: “Cryptic female Strawberry poison frogs experience elevated predation risk when associating with an aposematic partner”**

In this study we used an experiment to test if natural selection through predation could shape female preferences (and not only the degree of choosiness). We trained hens to peck on digitized images of frogs against a picture of a natural background on a screen and found that the estimated survival time of a cryptic frog was reduced when associating with an aposematic partner. Here we provide additional details of the methodology used in our experiment.

*Field data collection and screen calibration*

We used field data consisting of spectrum measurements and photographs of *Oophaga pumilio* frogs to construct test images and to calibrate the screen to suit the visual system of the used predator, the domestic fowl (*Gallus gallus domesticus*). Photographs, irradiance, and reflectance spectra data of frogs and habitat substrate were collected during November and December 2007, and between May and June 2008, in Bocas del Toro, Panamá. Irradiance and reflectance spectra of frog dorsal colour and habitat substrates were measured using a custom built teleradiometer consisting of an Avantes portable spectro-radiometer (AvaSpec-2048FT-SPU), connected with a 400-nm optic cable to a modified Nikon FM2 camera with a Nikon Rayfact PF10545MF-UV Quartz lens (Nikon Corporation, Tokyo, Japan) with a quartz filter. A Nikon HS-8 s (Nikon Corporation) lens shade was attached to the lens that was set for an aperture of 4.5 and a focus distance of 48 cm (Qvarnström et al. 2014). The same equipment was used to calibrate a 22-inch Samsung S22C200 computer screen to match the relative quantum catch by chicken cone photo receptors resulting from spectra measured in the field. The relative pair-wise photoreceptor difference (SWS2, MWS and LWS) in quantum catch was less than 6 % (for details see Qvarnström et al. 2014, supporting information). For this purpose the software Overture Version 1. 0. 1. © 2011 OceanOptics Inc. was used together with an R script developed by Håstad and Ödeen (2008).

*Experimental setup and training*

The experimental setup consisted of a skinner box (50 x 60 x 60 cm) with a smaller ’holding box‘ (35 x 35 cm) attached to one end, and a screen monitor attached to the other end (Fig S1). To let the experimenter have a full view of the inside of the skinner box, a camera was connected to an additional screen (Screen 2) on the experimenters desk was placed in one of the corners of the skinner box (Fig S1). The skinner box was separated by a sliding door from the holding box, this door was connected to a string mechanism that allowed the door to open or close when needed. To make certain that the hens could comfortably access the whole screen area where the images were shown, only an area of 477 mm width and 193 mm height of the computer screen was visible from the inside of the skinner box (sensu Qvarnström et al. 2014). Underneath the screen there was a sliding tray where the reward was delivered (a piece of spaghetti), which was opened and closed by the experimenter. The hen inside the skinner box could not see the experimenters while the experiment was underway.

The hens were very precise when pecking on the frogs, making it straight forward to determine whether they pecked correctly when viewing the camera footage. In cases when it was difficult for the observer to see the frog through the camera footage, the contour of the frog was drawn with a whiteboard marker on Screen 2.


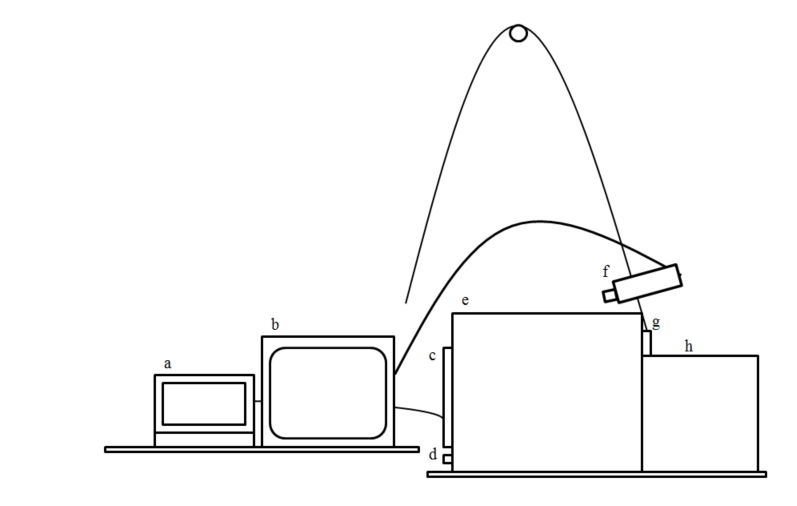


**Fig S1. Experimental setup.** (a) Computer connected to screen 1. (b) Screen 2 receiving camera footage. (c) Screen 1 showing experimental images. (d) Feeder. (e) Skinner box. (f) Camera. (g) Sliding door. (h) Holding box.

Each individual hen was placed in the holding box while preparing the reward tray and the image. Once the frog was spotted by the experimenter, the sliding door was opened and the latency until the hen pecked on the green frog recorded with a manual chronometer (catch time). If the hen failed to find the frog within 120 seconds, the trial was terminated. After the reward was given, the hen was gently herded back to the holding box to repeat the procedure. If the hen started to look uncomfortable or jumped out of the skinner box, the session was interrupted; this usually happened after an hour of continuous experimentation.

. In cases when it was difficult for the observer to see the frog through the camera footage, the contour of the frog was drawn with a whiteboard marker on Screen 2 (Fig S1).


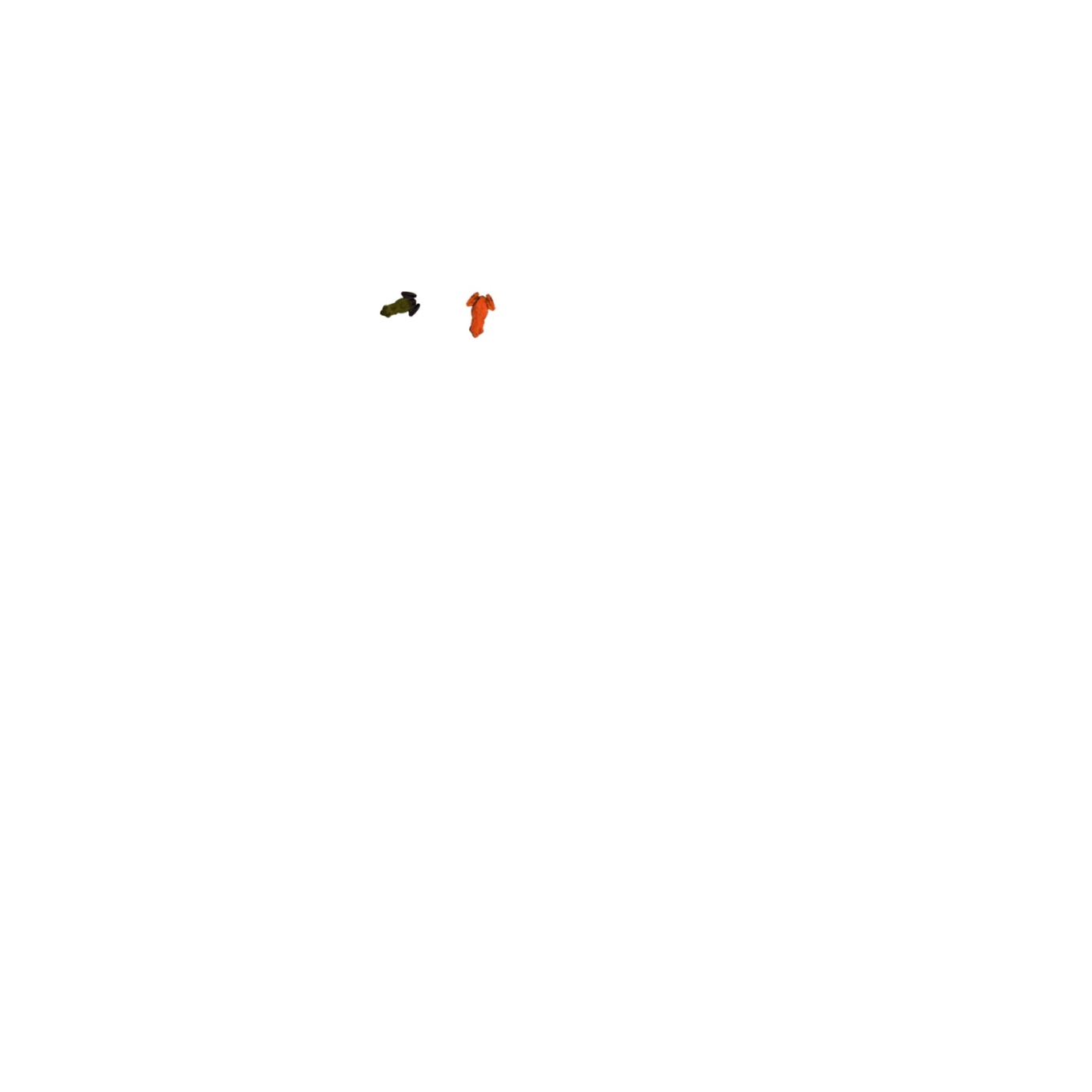

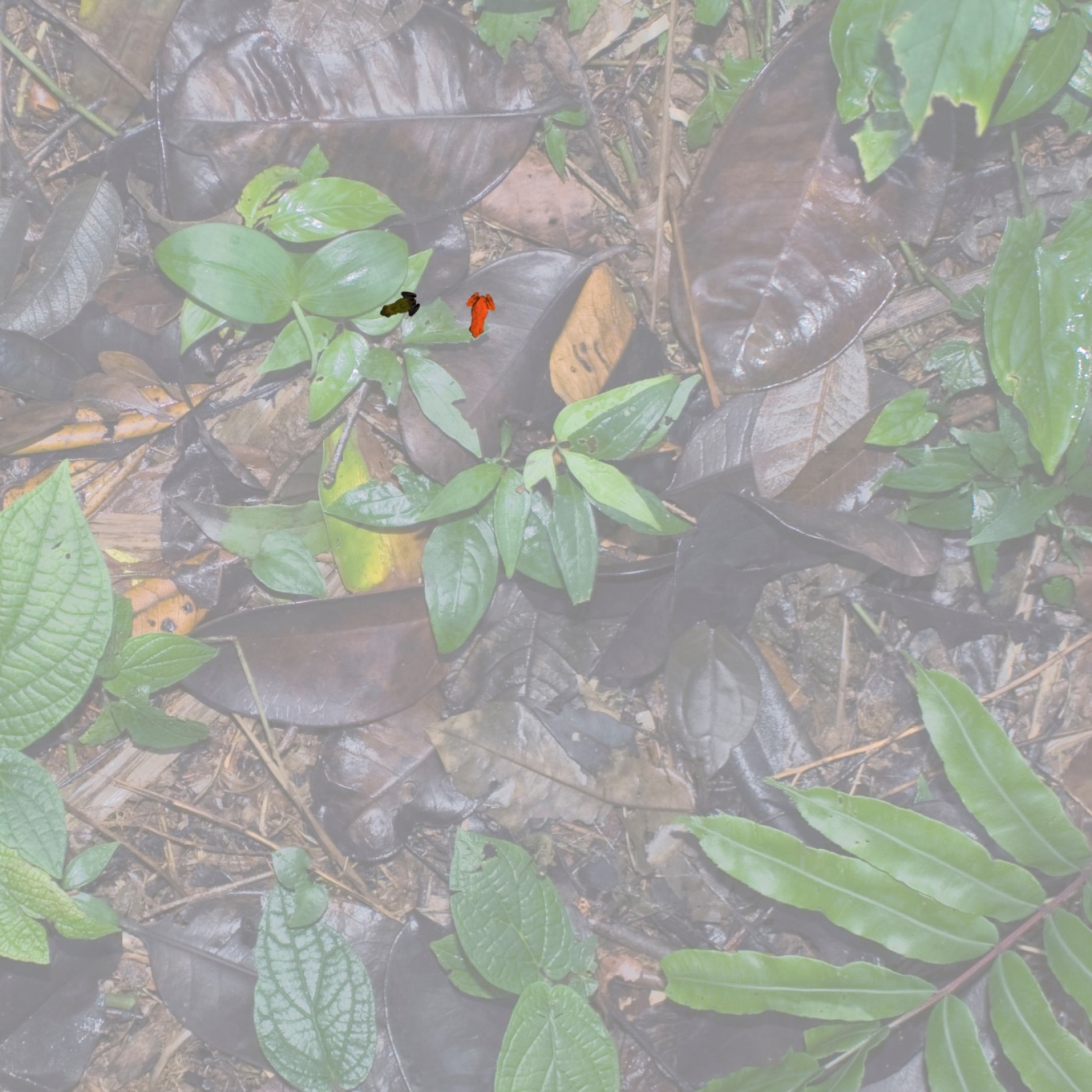

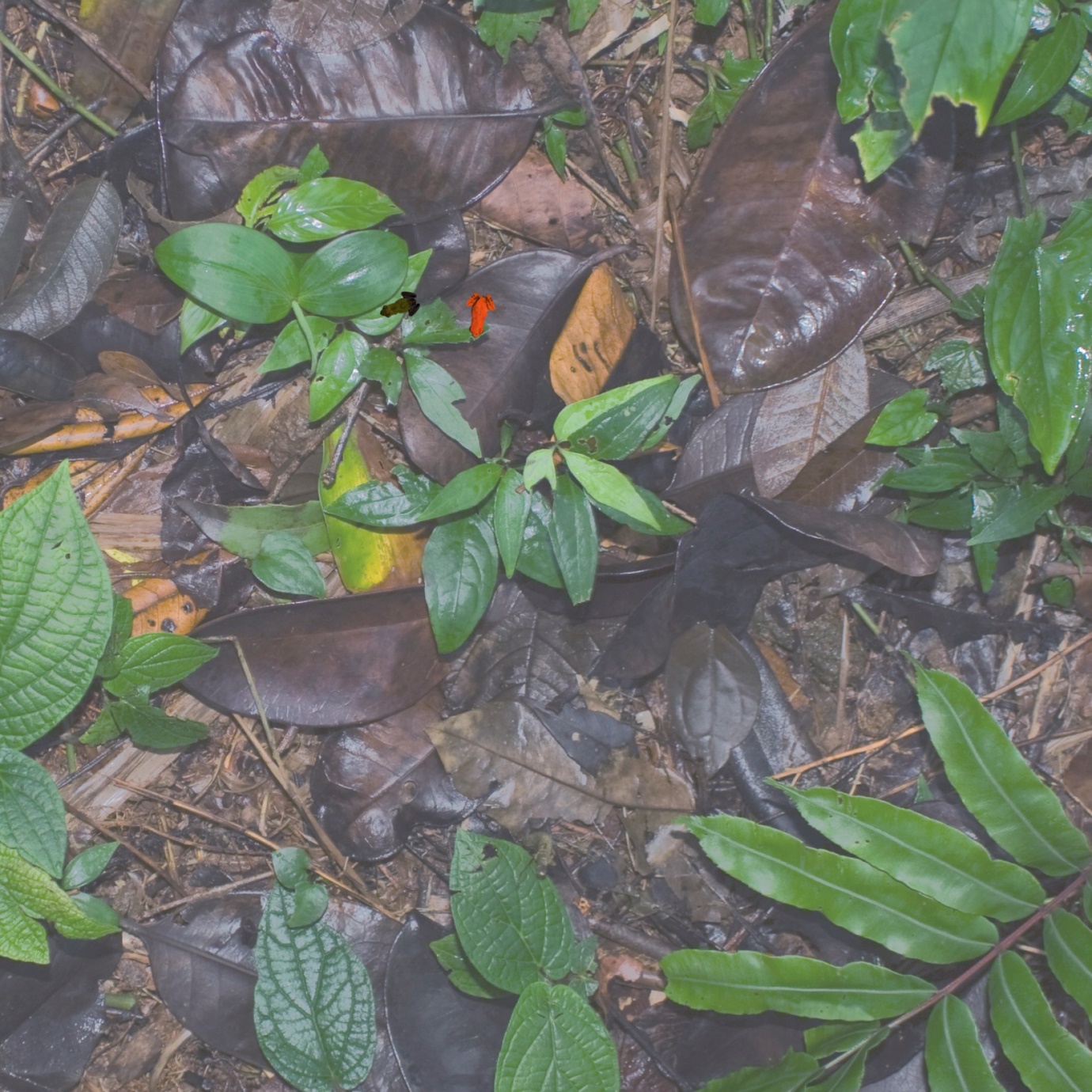

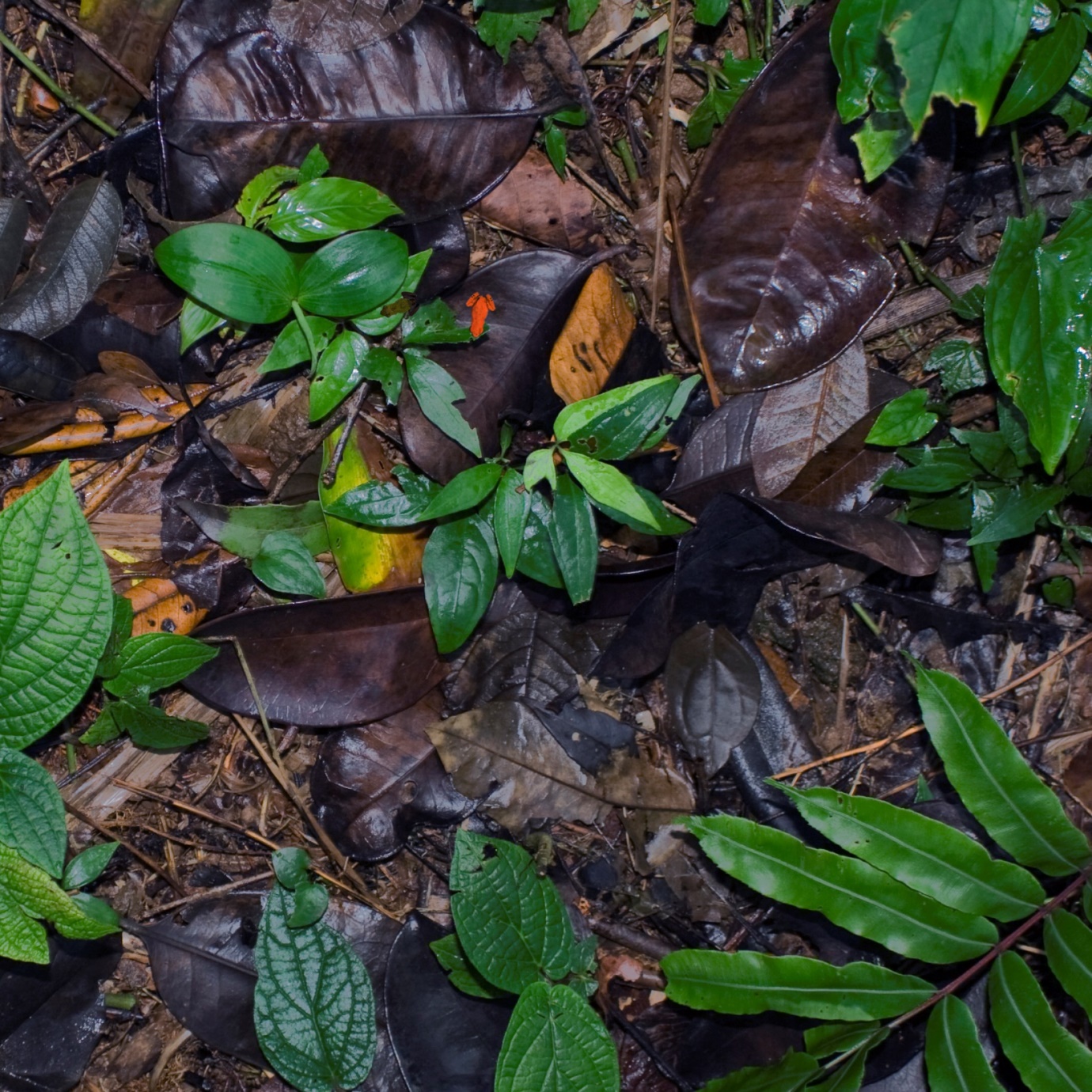


**Fig S2. Example of training images.** Starting from left to right there is a background saturation of 0%, 50%, 75% and 100%.

The treatments described on table 1 of the main manuscript were presented to the hens in a random order until the desired number of data points was reached (10 trials for each treatment for each bird).
